# Exploring the Impact of mRNA Modifications on Translation Efficiency and Immune Tolerance to Self-Antigens

**DOI:** 10.3390/vaccines12060624

**Published:** 2024-06-05

**Authors:** Mouldy Sioud, Asta Juzeniene, Stein Sæbøe-Larssen

**Affiliations:** 1 Department of Cancer Immunology, Oslo University Hospital, Radiumhospitalet, Ullernchausseen 70, 0379 Oslo, Norway; 2 Department of Radiation Biology, Oslo University Hospital, Radiumhospitalet, Ullernchausseen 70, 0379 Oslo, Norway; asta.juzeniene@rr-research.no; 3 Department of cellular Therapy, Oslo University Hospital, Radiumhospitalet, Ullernchausseen 70, 0379 Oslo, Norway; stein.saboe-larssen@rr-research.no

**Keywords:** mRNA vaccine, RNA modifications, pseudouridine, tumor antigens, immune tolerance

## Abstract

Therapeutic modified mRNAs are being developed for a broad range of human diseases. However, the impact of potential miscoding of modified mRNAs on self-tolerance remains unknown. Additionally, more studies are needed to explore the effects of nucleoside alkylation on translation. While all six tested modifications are tolerated as substrates by T7 RNA polymerase and inhibited mRNA immunogenicity, the translation efficiency varied significantly depending on the type of modification. In contrast to methylation, ethylation at the N1 position of pseudouridine (Ψ) hindered translation, suggesting that the C5-C1’ glycosidic bond alone is not a critical element for high translation. Inhibition of mRNA translation was also observed with 5-methoxyuridine modification. However, this inhibition was partially alleviated through the optimization of mRNA coding sequences. BALB/c mice immunized with syngeneic ψ-modified mRNA encoding for Wilms’ tumor antigen-1 (WT1) developed a low but significant level of anti-WT1 IgG antibodies compared to those immunized with either unmodified or N1-methyl ψ-modified mRNA. Overall, the data indicate that adding a simple ethyl group (-CH_2_CH_3_) at the N1 position of ψ has a major negative effect on translation despite its reduced immunogenicity. Additionally, mRNA containing Ψ may alter translation fidelity at certain codons, which could lead to a breakdown of immune tolerance to self-antigens. This concern should be taken into account during gene replacement therapies, although it could benefit mRNA-based vaccines by generating a diverse repertoire of antigens.

## 1. Introduction

The use of synthetic ribonucleic acid (RNA) to direct sequence-specific messenger (m) RNA degradation or express proteins is a promising therapeutic avenue, circumventing the safety concerns associated with viral or DNA-based strategies [1]. However, RNA faces degradation challenges, including extracellular ribonucleases and non-enzymatic degradation due to the 2′-hydroxyl group, potentially causing phosphodiester backbone cleavage [2]. To address these limitations, modified nucleosides have been incorporated into RNA to enhance stability [3,4]. For instance, replacing pyrimidine ribonucleosides, cytidine (C) and uridine (U), with their 2′-amino modified counterparts during in vitro transcription has led to the synthesis of nuclease-resistant catalytic RNAs, known as ribozymes [5,6]. Animals treated with these modified catalytic RNAs showed no adverse effects.

Over the last two decades, small interfering RNAs (siRNAs) technology has been extensively used as a way to study gene function and develop treatments for various diseases like cancers and virus infections [7,8]. However, certain lipid-formulated siRNA sequences can activate innate immunity, raising safety concerns about their use for therapeutic purposes [9]. Further studies showed that certain sequence motifs, particularly with high GU or U content, are important determinants in triggering the activation of pro-inflammatory cytokine and type I interferon pathways [10,11,12]. Both sense (mRNA sequence) and antisense strands of siRNAs activated innate immunity in peripheral blood mononuclear cells [12]. Additionally, siRNAs produced by phage T7 RNA polymerase, which adds a 5′ triphosphate to the transcripts, induced a strong type I interferon response [13]. This response was found to be mediated with retinoic acid-inducible gene-I (RIG-I), a cytoplasmic RNA sensor receptor expressed by most human cells [14]. With respect to TLR ligands, timely studies have shown that TLR7 and TLR8 recognize viral RNAs and GU-rich single-stranded RNA [15,16].

To mitigate innate immune activation by exogenous siRNAs, we have proposed the use of modified nucleosides [17]. With this regard, Morrissey and colleagues were the first to experimentally report that RNA methylation can suppress the immunostimulatory properties of siRNAs in vivo [18]. Unlike the unmodified siRNAs alone, the chemically modified siRNAs formulated in stable nucleic-acid-lipid particles did not induce interferon alpha and inflammatory cytokines in the serum of injected mice. With respect to RNA motifs, only uridines were identified as critical for Toll-like receptor (TLR) 8 activation in human monocytes, and their substitution with 2′-*O*-methyl-modified counterparts or adenosines inhibited innate immune activation [19]. Ribose 2′-*O*-methylation is one of the most common and widespread type of naturally occurring RNA modifications found in ribosomal RNA, transfer RNA, small nucleolar RNA, and messenger RNA. This modification can occur in all four nucleotides and other non-canonical nucleotides [20,21].

The risk of activating innate immunity complicates therapeutic applications of not only siRNAs, but also mRNA, where the therapeutic goal is to produce more proteins. Karikó and colleagues reported that endogenous RNA modifications, including 2′-*O*-methyluridine, 5-methyluridine (m^5^U), 5-methylcytidine (m^5^C), N6-methyladenosine (m^6^A), and pseudouridine (ψ) can reduce immunogenicity mediated by TLR3, TLR7, and TLR8 in HEK293 cells and human dendritic cells [22]. Further studies demonstrated that incorporating modified nucleosides such as ψ, N1-methylpseudouridine (m^1^ψ) and 5-methylcytidine (m^5^C) not only suppresses innate immune activation, but also enhances mRNA translation efficiency [23,24]. This breakthrough in RNA technology, coupled with the utilization of the ionizable lipid nanoparticle delivery systems [25,26], facilitated the rapid development of highly effective COVID-19 vaccines [27]. Other key features of modified mRNA-based therapeutics include minimal risk of genomic modification and rapid transient production of higher levels of therapeutic proteins when compared to DNA-based strategies [1].

To date, no study has investigated whether in vitro transcribed (IVT) mRNAs affect self-antigen tolerance due to potential translation errors with modified nucleosides. Additionally, it is uncertain whether alkylation at position N1 of pseudouridine (Ψ) would enhance translation, similar to the observed effects of methylation. By targeting two different mRNA sequences, here we demonstrate that all examined modifications at position 5 of uridine either hindered or failed to enhance translation. While ψ and m^1^ψ enhanced protein expression, ethylation at the N1 position of ψ severely blocked translation, highlighting the crucial role of the imino (NH) or methyl group at this position in maintaining and/or enhancing mRNA translation. The data also suggest that tolerance to self-antigens can be broken by ψ-modified mRNA

## 2. Materials and Methods

### 2.1. Nucleotides, Antibodies, and Enzymes

Modified nucleotides 5-methyluridine, 5-methoxyuridine, pseudouridine, N1-methyl speudouridine, N1-ethylpseudouridine, and anti-reverse cap (ARCA) were obtained from Trilink (San Diego, CA, USA). 5-hydroxylmethyluridine was obtained from APExBIO (Houston, TX, USA). T7 RNA polymerase, RQ1 RNase-free DNase, ribonucleotides (rATP, rGTP, rUTP, rCTP), Antartic phosphatase, and transcription buffer were obtained from Promega (Madison, WI, USA). Restriction enzymes and RNase inhibitor were obtained from New England BioLabs (Ipswich, MA, USA). Monoclonal antibodies against GFP and β-actin were obtained from Santa Cruz Biotechnology (Dallas, TX, USA). Monoclonal antibodies against Survivin and WT1 were obtained from Proteintech (Proteintech, Rosemont, IL, USA) and ThermoFisher Scientific (Waltham, MA, USA), respectively. Recombinant GFP protein was obtained from Novus Biologicals (Centennial, CO, USA). Other chemicals were obtained from Sigma-Aldrich (St. Louis, MO, USA).

### 2.2. Cell Culture

Human embryonic kidney 293T cells and HeLa cells were obtained from the American Type Culture Collection (Manassas VA, USA). Both cell lines were cultured in Dulbecco’s modified Eagle’s medium (DMEM) supplemented with 10% fetal bovine serum and antibiotics.

### 2.3. Plasmids

The pT7pA100 mRNA expression vector is a modified version of the pCIpA102 plasmid [28], by adding the sequence GGAGA to the end of the T7 primer sequence to obtain a canonical T7 promoter sequence (TAATACGACTCACTATAGGGAGA) and two additional restriction cut sites 3′ to the polyA region for linearization prior to in vitro transcription (https://www.plasmidtools.com (accessed on 15 September 2015)). PCR-amplified open reading frames encoding EGFP and Survivin were cloned into the pT7pA100 vector (pT7pA100-GFP and pT7pA100-Survivin), and the sequences was verified by Sanger sequencing (GATC Biotech). The coding sequences were flanked by the 5′ and 3′ SV40 untranslated regions. A synthetic gene containing the mouse WT1 open reading frame (P22561.1) was synthesized by GenScript and cloned into the EcoRI/NotI-digested pT7pA100 vector downstream of the T7 promoter sequence (pT7pA100-mWT1). Similarly, two synthetic genes containing optimized Survivin open reading frame or β-globin 5′ and 3′ UTRs, respectively, were synthesized by GenScript and cloned into EcoRI/NotI-digested pT7pA100 downstream of the T7 promoter (pT7pA100-SurCSO; pT7pA100-Sur-βg). For sequence information, see the supplemental file. Positive clones were selected and verified by DNA sequencing. Plamids were isolated from the bacterial stocks using the Nucleospin plasmid isolation kit (Macherey-Nagel, Düren, Germany). DNA templates were linearized using the MfeI restriction enzyme, checked on 1% agarose gels, and then purified by adding 1/10 volume of 3M sodium acetate and 2.5 volumes of 100% ethanol. After incubation at −20 °C for 2 h, samples were centrifuged at 14,000× *g* at 4 °C for 30 min. DNA pellets were washed with 70% ethanol, air-dried, and then resuspended in UltraPure nuclease-free sterile water for subsequent in vitro transcription. DNA concentrations were determined using UV/Vis spectroscopy (NanoDrop 1000 Spectrophotometer).

### 2.4. In Vitro Transcription

The mRNAs were synthesized by in vitro transcription (IVT) using T7 RNA polymerase, starting with a linear DNA template (1 μg/100 µL reaction) containing the open reading frames of GFP, Survivin, or WT1 downstream of the T7 promoter sequence, as previously described [29]. All nucleotides were used at a final concentration of 4 mM in the transcription reaction, except for GTP, which was reduced to 1 mM to enhance capping efficiency. During transcription, the mRNAs were capped using ARCA (5 mM), included in the master reaction mix. To minimize variation between samples, all reagents were included in the transcription master mix, except for the modified nucleotides and uridine. The chaotropic agent urea was added to each transcription reaction at 0.8 M final concentration as described by Piao et al. [30]. A 100-nucleotide-long polyA tail, encoded directly in the DNA template, was added to the mRNAs. IVT mixtures were incubated at 37 °C for 3 h. After transcription, RQ1 RNase-free DNAase I was added to remove the DNA template, followed by treatment with Antarctic phosphatase to remove the triphosphate group from the 5′-ends of potential uncapped mRNAs. Subsequently, IVT mRNAs were purified using MEGAclear kit following the manufacturer’s instructions (Themo Fisher Scientific, Waltham, MA, USA). The concentrations of the IVT-mRNAs were determined using UV/Vis spectroscopy (NanoDrop 1000 Spectrophotometer), and the integrity of transcripts was analyzed by 1% agarose gel electrophoresis.

### 2.5. Agarose Gel Electrophoresis

After each transcription, an equal volume of crude IVT mRNA was loaded onto a 1% agarose gel to visually assess mRNA yield. Each gel was run in 1xTAE buffer at 120 V for 30 min. Gels were pre-stained with GelRed (Biotium, Fremont, CA, USA) and imaged with Biorad ChemiDoc XRS+ Imaging System.

### 2.6. Transfection

HEK293T cells and HeLa cells (5 × 10^5^) were seeded in 6-well plate for 24 h before transfection. The next day, the medium was replaced with fresh 2 mL complete medium before transfection with test molecules. A transfection mix was made by diluting the appropriate IVT-mRNA (250 ng, 500 ng, or 750 ng) in 250 μL OPTI-MEM and 5 µL LipoFectMax in 250 μL OPTI-MEM, which was incubated separately in room temperature for 5 min, before solutions were mixed and incubated for an additional 30 min at room temperature. After, the mixtures (0.5 mL in total/well) were added to the cells and incubated for 24 h at 37 °C before analysis by Western blotting. In the case of GFP, the cells were washed with PBS and IVT mRNA expression efficiency was measured by the number of GFP+ cells (positive cells on FITC channel in Canto II cytometer). Analysis was conducted on a BD FACS Canto II utilizing BD FACSDiva™ 6.1.3 software (BD Biosciences, San Jose, CA, USA). The collected data were then examined using FlowJo version 7.6.1 (FlowJo LLC, Ashland, OR, USA).

### 2.7. Cell Viability

HEK293T cells and HeLa cells in 96-well plate cells at a density of 2 × 10^5^ cells per 200 µL complete RPMI 1640. The next day, the cells were transfected with different doses of in vitro transcribed (IVT) mRNAs formulated in LipoFectMax transfection reagent. After 24 h, cell viability was subsequently evaluated using CellTiter 96^®^ AQueous One Solution Reagent (Promega, Madison, WI, USA) following the manufacturer’s instructions. Optical absorbance was recorded at 492 nm using a Sunrise microplate reader (TECAN, Switzerland) in conjunction with Magellan™ software version V7.2 SP1 (TECAN, Switzerland). The values obtained from cells transfected solely with LipoFectMax reagent (mock control) were considered as 100% for comparative analysis.

### 2.8. Western Blot Analysis

After transfection, cells were collected, washed with PBS buffer and then cytoplasmic protein extracts were prepared by incubating the cells in 50–100 μL lysis buffer (PBS supplemented with 1% NP-40, 1:100 protease inhibitor) for 30 min on ice with vortexing every 10 min. After, samples were centrifuged at 16,000× *g* for 20 min at 4 °C and protein concentrations within the supernatants were determined with the Bradford protein assay (BioRad, Irvine, CA, USA). Equal amounts of protein (30–40 μg/lane) were subjected to analysis via 10% or 12% SDS-PAGE, followed by the electrotransfer of proteins onto nitrocellulose membranes. Subsequently, the membranes were blocked with TBS-T buffer (20 mM Tris-HCl, 150 mM NaCl, and 0.1% Tween-20, pH 7.4) containing 5% non-fat milk or BSA at room temperature for 1 h. The membranes were then exposed to primary antibody, diluted in TBS-T supplemented with 1% BSA or non-fat milk, overnight at 4 °C. The following day, the membranes underwent washing with TBS-T three times (each wash lasting 10 min) and then incubated with secondary antibody diluted in TBS-T supplemented with 1% BSA or non-fat milk at room temperature for 1 h. Post three washes with TBS-T, the membranes were developed utilizing Clarity Western ECL Substrate (BioRad, Irvine, CA, USA), according to the manufacturer’s instructions. Visualization of the membranes was carried out using ChemicDoc™ MP Imaging System (BioRad, USA). Quantification analysis of proteins was calculated relative to β-actin using Image Lab™ 4.1 Imaging System (Bio-Rad, Irvine, USA).

### 2.9. Induction of Cytokines by the In Vitro Transcribed RNAs

Freshly isolated peripheral blood mononuclear cells (PBMCs) were seeded in 96-well plates (Nunclon™ Delta Surface, Thermo Fisher, USA) at a density of 2 × 10^5^ cells per 200 µL complete RPMI 1640 and stimulated with different doses of in vitro transcribed (IVT) mRNAs formulated in LipoFectMax transfection reagent. A transfection mix was prepared by diluting the appropriate amounts of IVT-mRNA (50 ng, 150 ng, and 250 ng) in 25 µL OPTI-MEM, and 1 µL LipoFectMax in 25 µL OPTI-MEM, which were incubated separately at room temperature for 5 min. Afterward, the solutions were combined (approximately 50 µL in total) and further incubated for an additional 30 min at room temperature. Subsequently, the mixtures were added to the cells and incubated for 24 h at 37 °C in a humidified atmosphere containing 5% CO_2_. Following the incubation period, culture supernatants were collected and analyzed for TNF-α and IFN-α contents using the BD OptEIA™ Human TNF ELISA set (BD Biosciences, USA) and Human IFN-α ELISA KIT (PBL Biomedical Laboratories), respectively. Absorbance readings were taken using a Sunrise microplate reader (TECAN, Switzerland) in conjunction with Magellan™ software (TECAN, Switzerland).

### 2.10. Purification of His-Tagged WT from HEK293T Cells

HEK293T cells were seeded onto cell culture flask T-75 to obtain cells that are 70–80% confluent the next day. Subsequently, the cells were transfected with IVT mRNAs (5 μg/flask) using LipoFectMax transfection reagent according to the manufacturer’s protocol. The cells were harvested by gentle scraping 24 h after transfection, washed in PBS buffer and protein lysates were prepared for His-tagged protein purification using MagneHis Protein purification System (Promga, Madison, WI, USA), as described by manufacturer’s instructions.

### 2.11. Animals

Female BALB/cJRj mice (6 weeks old) were purchased from Janvier Labs (Le Genest-Saint-Isle, France). After their arrival, mice were given a minimum of one week to adjust to the new environment before being used in the experiments. Animal studies were carried out in agreement with the protocols approved by the Institutional Committee on Research Animal Care and the Norwegian Food Safety Authority (FOTS-18095).

### 2.12. Immunization

Female BALB/c mice were divided into 5 groups (n = 5 per group) and inoculated with different mRNA formulations in LipoFectMax Transfection Reagent. Each mRNA vaccine was administered subcutaneously at 10 μg/mouse formulated in LipoFectMax and Opti-MEM Reduced Serum (total volume 100 μL) supplemented with 10 μL of complete Freund’s Adjuvant (CFA) (Sigma-Aldrich, St. Louis, MO, USA). CFA was used only for the primary immunization to boost antibody production. During the second and third immunization, we relied on the potential adjuvant effect of the lipid carrier. Notably, lipid nanoparticles used in vaccines have demonstrated potent adjuvant activity and inflammatory responses in both mice and humans [31,32]. Animals received three IVT mRNA vaccinations (one/week), and on week 4, mice were sacrificed, blood samples were taken from the heart, and processed serum samples were stored at −20 °C until analysis by ELISA. During vaccination, no significant body-weight reduction was observed in immunized mice compared to those that received only saline. Additionally, no ulceration at the injection sites was observed.

### 2.13. Analysis of WT1-Antibody Response by ELISA

Antibody response against mouse Wilms’ tumor protein 1 (WT1) protein variants was measured by ELISA using purified recombinant proteins as coating antigens and serum samples obtained from mice following immunization. Briefly, Costar 96-well high binding surface plates were coated overnight with 10 μg/mL WT1 in PBS and subsequently blocked for 1 h with 2% BSA in PBS. After washing, plates were incubated with serum samples diluted at 1/10 in PBS containing 1% BSA and 0.02% Tween 20 (assay buffer). Subsequently, plates were washed 3 times with assay buffer and further incubated for one hour at room temperature with alkaline phosphatase-conjugated goat-anti mouse IgG diluted at 1:2500 in assay buffer. After washing, the substrate for alkaline phosphatase (100 μL) was added to each well, and optical density (OD 405 nm) was measured using a Sunrise microplate reader (TECAN, Switzerland) together with Magellan™ software (TECAN, Switzerland). Similarly, serum antibodies to commercially available GFP protein were measured by ELISA.

### 2.14. Statistics

The data of independent experiments were summarized and displayed as mean plus/minus standard deviation. The difference between control and treated samples was analyzed using student’s *t*-test. One-way analysis of variance (ANOVA) was used for multiple comparisons. Statistical analysis was carried out using GraphPad Prism 4.0 software. A value of *p* < 0.05 was considered statistically significant

## 3. Results

### 3.1. T7 RNA Polymerase Tolerates Modifications at the C5 Position of Uridine or the N1 Position of ψ

**Figure 1:**
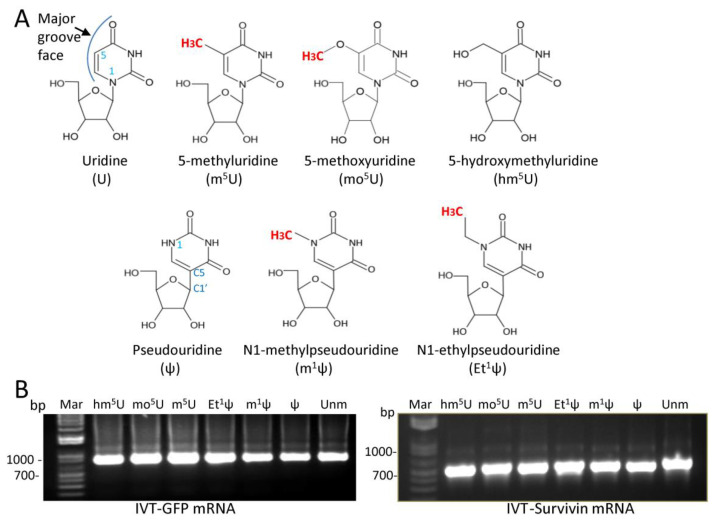
In vitro transcription: (**A**) Chemical structures of the modified nucleosides. The methyl group is highlighted in red. (**B**) Analysis of transcribed mRNAs on non-denaturing 1% agarose gels. Following transcription and treatment with DNAase to remove the DNA template, 5 µL from each transcription reaction was separated on a non-denaturing 1% agarose gel pre-stained with GelRed nucleic acid gel stain and visualized under ultraviolet illumination. The data are representative of several experiments. Unm = Unmodified; Mar = Marker.

First, we investigated whether specific nucleoside modifications, particularly Ψ alkylation, could selectively affect protein expression. The modifications chosen for this study were m^5^U, 5-methoxyuridine (mo^5^U), 5-hydroxylmethyluridine (hm^5^U), ψ, m1ψ, and N1 ethyl ψ (Et^1^ψ). All these modifications are located in the major groove face of nucleosides, and their chemical structures are shown in Figure 1A. As working models, mRNAs encoding for green fluorescence protein (GFP) and Survivin, a universal tumor antigen, were tested. First, T7 phage RNA polymerase was used to transcribe mRNA with modified nucleosides. Under our experimental conditions, all modified nucleosides were tolerated as substrates by T7 RNA polymerase, resulting in full-length GFP or Survivin transcripts containing uridine, ψ, m^1^ψ, Et^1^ψ, m^5^U, mo^5^U, or hm^5^U, (Figure 1B). No major differences were observed in signal intensities of IVT mRNA equipped with the various modifications, and comparable transcription patterns were consistently observed across IVT-mRNA samples from several independent syntheses. Of note, we uniformly incorporated a 5′ ARCA (Anti-Reverse Cap) as a standard cap structure in the synthesis of IVT-mRNAs, and all transcriptions were performed in the presence of 0.8 M urea, which has been shown to significantly reduce the formation of double-stranded RNA during transcription [30].

### 3.2. All Tested Modifications at the C5 Position of Uridine and the N1 Position of ψ Inhibited Innate Immune Activation

**Figure 2:**
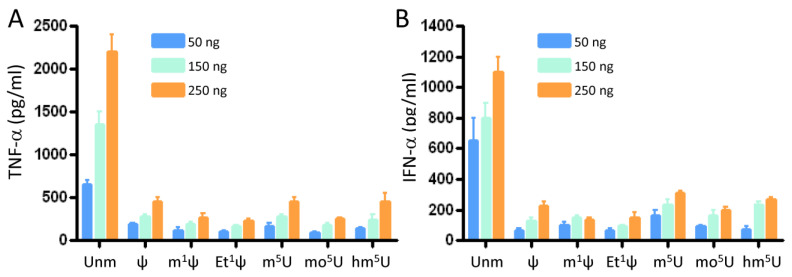
TNF-α and IFN-α production by IVT-mRNA transfected PBMCs. Freshly isolated human peripheral blood mononuclear cells (PBMCs) were seeded in a 96-well plate at a density of 2 × 10^5^ cells per well in 200 µL of complete medium. The cells were then transfected with either unmodified (Unm) or modified IVT GFP mRNA, which was formulated using LipoFectMax transfection reagent (50 μL total). Three different doses (50, 150, 250 ng/well; corresponding to 0.2 ng/μL, 0.6 μg/μL; 1 ng/μL) were tested. After 24 h, the levels of TNF-α (**A**) and IFN-α (**B**) in the culture supernatants were measured using ELISA. The presented values represent the means ± standard deviation (SD) of triplicate measurements. This data are representative of three independent experiments.

Unmodified therapeutic RNAs, including ribozymes, siRNAs, and IVT mRNAs, are considered to be immunostimulatory, triggering the secretion of various pro-inflammatory cytokines and type I interferons [12]. Aggressive response of the immune system to exogenous RNA can lead to unwanted or even detrimental side effects causing degradation of therapeutic mRNAs. To assess the immunogenicity of IVT mRNAs, freshly isolated peripheral blood mononuclear cells (PBMCs) were transfected with IVT mRNA at three different doses using LipoFectMax transfection reagent. The secretion of tumor necrosis factor alpha (TNF-α) and interferon alpha (IFN-α) was measured at 24 h post-transfection using DuoSet ELISA kits (Figure 2A,B). Unmodified IVT GFP mRNA induced the expression of TNF-α, and IFN-α. All modified IVT-mRNA resulted in significantly low levels of TNF-α and IFN-α at three tested mRNA doses. ψ, m^5^U, and hm^5^U modifications induced TNF-α and IFN-α expression at high concentrations, albeit significantly lower than unmodified mRNAs. There were no significant differences between TNF-α and IFN-α levels from PBMC transfected with m^1^Ψ and Et^1^Ψ IVT mRNAs.

### 3.3. Ethylation at the N1 Position of ψ Abrogates Translation in Human Cells

**Figure 3:**
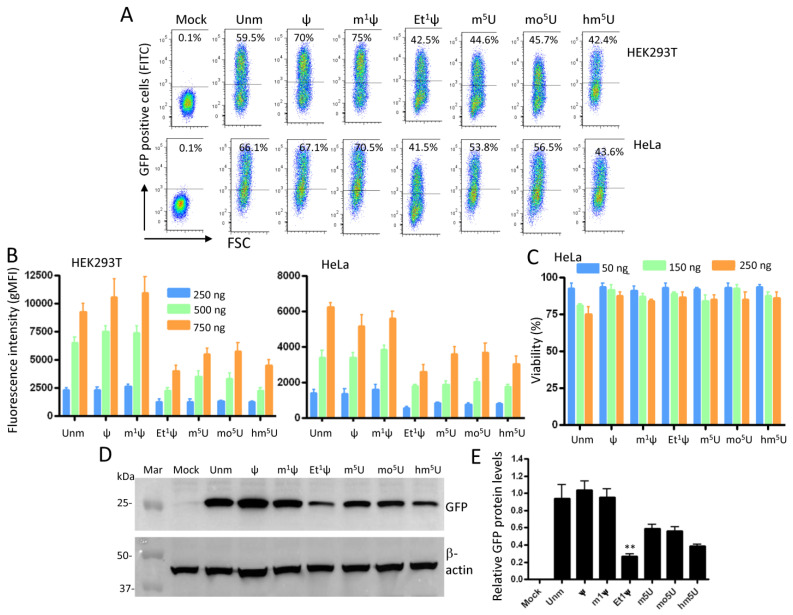
Impact of mRNA modifications on GFP expression. Cells were seeded in a 6-well plate at a density of 5 × 10^5^ cells per well in 2 mL of complete medium, followed by transfection with three different dosages of IVT mRNA (250, 500, and 750 ng/well) formulated in LipoFectMax transfection reagent (0.5 mL total) as described in the Materials and Methods. The final concentrations of the tested molecules were 0.1 ng/μL, 0.2 ng/μL, or 0.3 ng/μL: (**A**) Representative flow cytometric density plots displaying GFP expression in HEK293T cells and Hela cells 24 h post-transfection (500 ng dose is depicted). (**B**) GFP expression levels were quantified by flow cytometry and plotted as geometric mean fluorescence intensity (gMFI) ± standard deviation (SD). The tested doses are indicated, and error bars represent SD from four independent experiments. (**C**) Cell viability. HeLa cells were seeded in 96-well plate at a density of 1 × 10^5^ cells per well in 200 μL of medium, followed by transfection with three different dosages of IVT mRNA (50 ng, 150 ng, and 250 ng). After 24 h, cell viability was determined using the CellTiter 96R Aqueous One Solution reagent. The results are expressed as mean of percentage values of triplicate measurements ± standard deviation and are representative of three independent experiments. The values obtained from cells transfected solely with LipoFectMax reagent (Mock) were considered as 100%. (**D**) A representative example of Western blot analysis depicting GFP expression in HEK293 cells transfected with either unmodified (unm) or modified IVT mRNA (500 ng dose). Protein lysates were prepared 24 h post-transfection. To ensure consistent protein loading, the same membrane was probed with a monoclonal antibody against β-actin. The blot shown is representative of at least four independent experiments. (**E**) GFP expression levels (n = 3) were quantified by densitometry and normalized to β-actin expression. Both protein signals were determined using the ImageJ program on less exposed membranes to avoid signal saturation. ** *p* < 0.01. Unm = unmodified; Mar = marker.

To assess translation efficiency, IVT mRNA coding for natural GFP (with non-optimized codon sequence) was transfected into either human HEK293T or HeLa cells, and protein expression was analyzed by flow cytometry. Representative flow cytometry dot plots of transfected cells (500 ng dose) are shown in Figure 3A. Around 40% to 75% of the cells expressed GFP protein, albeit with varying fluorescence intensities. Bar graphs display geometric mean of fluorescence intensity (gMFI) for the three IVT mRNA concentrations determined by flow cytometry over 24 h transfection time (Figure 3B). The results represent the average of four independent experiments. Compared to unmodified mRNA, mean expression was comparable for GFP mRNAs containing ψ, and m^1^ψ, but lower for Et^1^ψ, mo^5^U, m^5^U, and hm^5^U. Notably, all three modifications at position 5 of uridine (m^5^U, mo^5^U, hm^5^U) reduced protein expression. This outcome was consistent for four different mRNA syntheses. Unlike the methyl group, replacement of N1-H atom of ψ with an ethyl group inhibited translation. This finding suggests that the presence of the C5-C1’ bond, a major intrinsic characteristic of pseudouridylation enabling the nucleobase to rotate freely around the sugar ring [33], may not account for the high protein expression observed with ψ and m1ψ. Under our experimental conditions and the tested mRNA doses, cell viability was not significantly affected by modified mRNAs (Figure 3C, as a representative example).

In addition to flow cytometry analysis, the expression of GFP protein was evaluated with Western blotting (Figure 3D, a representative blot). The data show that GFP protein of expected size was produced by all chemically modified IVT mRNAs. The relative level of protein expression was obtained by comparing the expression of GFP to that of β-actin from three independent experiments (Figure 3E). Overall, the protein expression levels align with those obtained with flow cytometry, and the lowest level of GFP protein was observed for the Et^1^Ψ modification (≈3.5-fold reduction, *p* < 0.01). In agreement with recent reports [34,35], we did not see increase in protein expression with mo^5^U modification as reported early [36].

**Figure 4:**
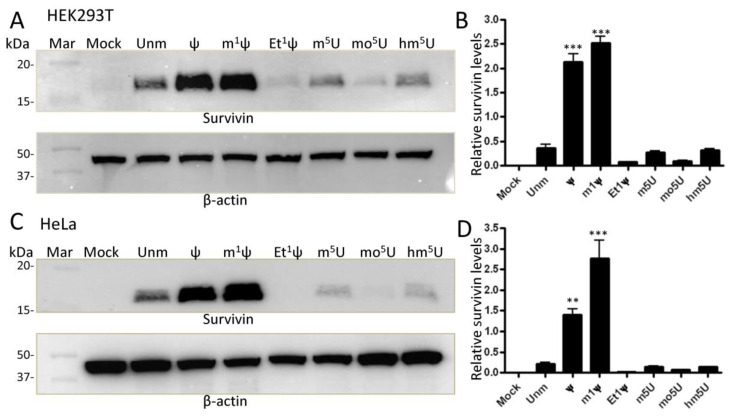
Impact of mRNA modifications on Survivin expression. HEK293T cells (**A**) and HeLa cells (**C**) were seeded in a 6-well plate at a density of 5 × 10^5^ cells per well in 2 mL of medium. They were then transfected with either unmodified (unm) or modified IVT mRNAs (500 ng dose) for 24 h, followed by preparation of protein lysates for analysis by Western blotting. To ensure consistent protein loading, the same membranes were probed with an antibody against β-actin. Each blot shown is representative of three independent experiments. (**B**,**D**) Survivin expression levels (n = 3) were quantified by densitometry and normalized to β-actin expression per lane. Both protein signals were determined using the ImageJ program on less exposed membranes to avoid signal saturation. ** *p* < 0.01; *** *p* < 0.001. Mock = transfected with lipids only; Unm = unmodified; Mar = marker.

To ensure that the observed effects, especially with Et^1^Ψ modification, were not specific to the sequence of GFP mRNA, we investigated the expression of modified IVT mRNAs encoding for Survivin, a universal tumor-associated antigen (TAA) included in our mRNA-based dendritic cell cancer vaccination program at Oslo University Hospital [29,37]. HEK293T cells were transfected with natural modified IVT mRNA for 24 h, and protein extracts were prepared and analyzed by Western blotting using antibody-specific for Survivin (Figure 4A,B, as representative examples). Natural Survivin mRNA exhibited high expression with ψ and m1ψ (≈7.5-fold and 9-fold increase, respectively), but very poor expression with Et^1^ψ and mo^5^U compared to the unmodified version (≈4.5-fold reduction). Notably, the enhancement of protein expression afforded by ψ was totally abrogated by ethylation. Similar trends were observed for Survivin mRNA in Hela cells, with Ψ and m1Ψ yielding around a 6- and 11-fold increase in expression, respectively, than U (Figure 4C,D as representative examples). In Hela cells, m^1^ψ outperformed ψ and Et^1^ψ totally inhibited protein expression.

### 3.4. Sequence Optimization Does Not Rescue the Inhibitory Effect of N1 Ethylation of ψ on Translation

**Figure 5:**
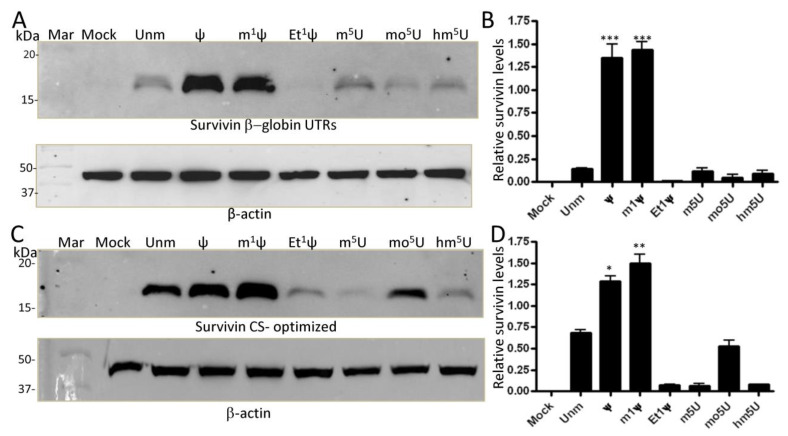
Impact of mRNA optimization on Survivin expression. HeLa cells were seeded in a 6-well plate at a density of 5 × 10^5^ cells per well in 2 mL of medium. Cells were transfected with either β-globin UTRs (**A**) or codon sequence (**C**) optimized IVT Survivin mRNA containing different nucleoside modifications (500 ng dose) for 24 h and then protein lysates were prepared and analyzed by Western blotting. Each blot shown is representative of at least three independent experiments. Survivin signals (n = 3) were normalized to that of β-actin (**B**,**D**). * *p* < 0.05; ** *p* < 0.01; *** *p* < 0.001. Mock = transfected with lipids only, Unm = unmodified; Mar = marker.

To enhance translation efficiency, many mRNA therapies utilize untranslated regions (UTRs) from highly expressed human genes, such as α- and β-globins [38,39]. Similarly, codon optimization is commonly employed to increase protein expression [40]. Building upon these strategies, we investigated the potential impact of sequence optimization on protein expression. To this end, we generated two Survivin mRNA sequences: one featuring an optimized coding sequence designed using the GenSmart™ Codon Optimization algorithm (GenScript), and the other incorporating the 5′ and 3′ β-globin UTR sequences along with the natural coding sequence (not optimized). Replacement of the initially used SV40 UTRs with those of β-globin did not significantly alter protein expression profile in HeLa cells, except that m1Ψ did not show superior performance (Figure 5A,B, as representative examples). Once again, only Ψ and m^1^Ψ boosted protein expression (≈8.5-fold increase). Optimization of the codon sequence resulted in enhanced translation of both unmodified and mo^5^U-modified mRNA, yet it failed to counteract the inhibitory effects of Et^1^Ψ, m^5^U, and hm^5^U modifications (Figure 5C,D, as representative examples). Under our experimental conditions, Ψ and m1Ψ led to a 0.7-fold and a 1.2 fold increase in protein expression, respectively. Overall, all Survivin sequence variants containing ψ and m^1^ψ expressed very well in both HEK293T cells and HeLa cells. However, all Et^1^ψ variants expressed at extremely very low levels. Hence, the presence of an ethyl group –CH_2_CH_3_ at the N1 position of ψ is detrimental to translation despite its reduced immunogenicity.

### 3.5. Ψ-Modified mRNA Vaccine Induces Antibody Responses to Syngeneic Wilms’ Tumor Antigen 1

**Figure 6:**
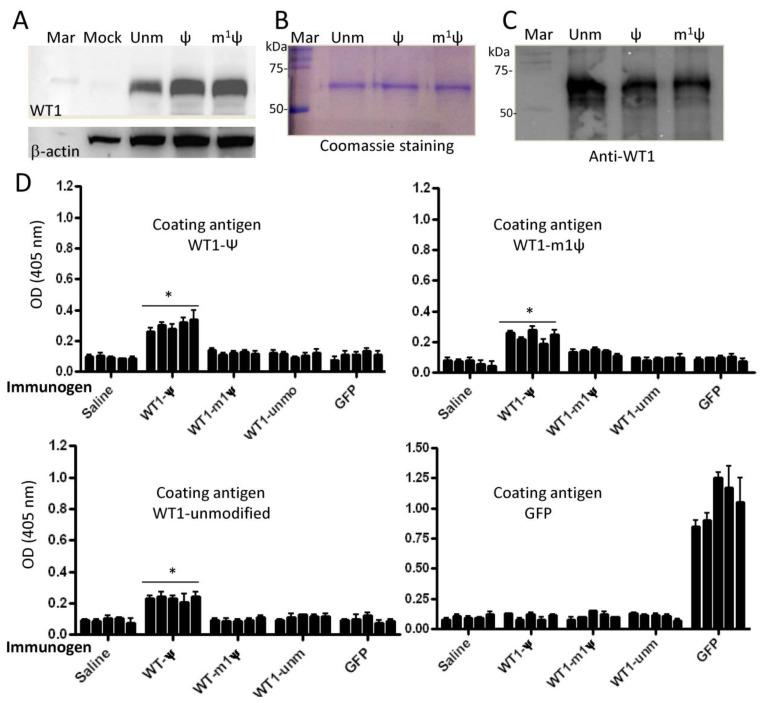
Analysis of mouse WT1 mRNA expression and induction of antibody responses: (**A**) Expression of mouse WT1 IVT mRNA in HEK293T cells. Cells were transfected with unmodified or modified IVT mRNA (500 ng dose) for 24 h, and then gene expression was analyzed by Western blotting. (**B**) His-tagged mouse WT1 expressed in HEK293T cells was purified using the MagneHis protein purification kit, and approximately 0.5 µg from each preparation was analyzed by 10% SDS-PAGE followed by Coomassie staining. (**C**) Purification of WT1 was confirmed by Western blotting using an anti-mouse WT1 monoclonal antibody. (**D**) Humoral immune response in BALB/c mice (n = 5/group) after immunization with unmodified (unm), ψ-, or m1ψ-modified IVT mRNA encoding mouse WT1 (self-antigen). As a positive control, a group of mice were immunized with unmodified IVT mRNA coding for GFP (foreign antigen). Serum samples were collected one week after the third immunization and tested for the presence of WT1-specific antibodies by ELISA using recombinant WT1 protein variants or GFP protein as coating antigens. Results of triplicate measurements are expressed as mean density (405 nm) ± standard deviation (SD) at a serum dilution of 1:10. The data are representative of three independent ELISA measurements. Each column represents a mouse. * *p* < 0.05. Mock = transfected with lipids only; Unm = unmodified; Mar = marker.

Based on previous studies on tRNAs, it is evident that modified nucleotides can alter the fundamental properties of RNAs, including their secondary structures, base stacking and pairing abilities, and translation fidelity [41,42,43]. Hence, it has been proposed that certain mRNA modifications may promote the incorporation of multiple amino acids on a single codon, as demonstrated for the unusual asparagine codon AAA [44]. Some studies indicate that this is indeed possible with Ψ [45,46]. Notably, the introduction of even one amino acid substitution can directly affect the structure and immunogenicity of the translated protein, as documented in cancers [47]. To test whether Ψ could break tolerance to self-antigens, we utilized the wild-type mouse Wilms’ tumor antigen 1 (WT1), a self-antigen highly conserved between humans and mice. WT1 is known to be over-expressed in various human malignancies and is a key antigen of our mRNA-based DC cancer vaccine program [48,49]. First, we confirmed the expression of ψ- and m^1^ψ-modified mRNA in HEK293T cells (Figure 6A). Both modifications enhanced mRNA translation when compared to unmodified mRNA. Second, groups of BALB/c mice were subcutaneously immunized with various LipoFectMax-formulated IVT mRNAs as described in Materials and Methods. One week after the third immunization, sera were collected and tested for antibodies against the WT1 or GFP protein. For these experiments, His-tagged mouse WT1 protein expressed in HEK293T cells was purified by Ni2+ resin chromatography and then used to coat ELISA plates. The purity of WT1 protein prepared from cells transfected with unmodified (unm), ψ-, or m^1^ψ-modified mRNAs is shown in Figure 6B. The purification of WT1 protein was further validated using a specific monoclonal antibody in Western blotting (Figure 6C). Immunization of BALB/c mice with ψ-modified WT1 antigen-induced detectable antibody titers against WT1 protein in BALB/c mice (Figure 6D, first panel, *p* < 0.05). Notably, the sera of animals vaccinated with ψ WT1 mRNA cross-reacted with WT1 protein purified from HEK293T cells transfected with either unmodified or m1ψ WT1 mRNA. No significant antibodies were measured in mice immunized with unmodified mRNA. Sera from mice immunized with m^1^ψ mRNA showed a slight increase in antibody response toward WT1 protein purified from HEK293T cells transfected with m^1^ψ modified mRNAs as compared to controls (Figure 6D, panel upper right). However, this response was not significant. As expected, unmodified GFP mRNA (foreign protein) induced a strong antibody response to commercially available recombinant GFP protein (Figure 6D, last panel). Overall, the data suggest that potential translation alterations in WT1 protein expressed from ψ-modified mRNA can break tolerance to self-antigens. Alternatively, pseudouridine may result in a cytokine or leukocyte environment, or in a manner of antigen presentation that is particularly effective at breaking immune tolerance. Optimizing the vaccination protocol by testing various lipid carriers, including ionizable nanoparticles, both with and without adjuvants approved for human vaccines, should facilitate the translation of the current findings to clinical practice.

## 4. Discussion

Modified nucleosides are utilized to prevent the activation of innate immunity by IVT mRNAs. Certain modifications, such as ψ, m^1^ψ, mo^5^U, and m^5^C, have been shown to increase translation efficiency. However, this was not always found to be the case, and some questions related to the induction of immunity to the encoded therapeutic proteins remain unanswered. The present study provides the first evidence that Ψ-modified mRNA treatment can induce an antibody response to self-antigens and that the addition of a simple ethyl group at the N1 position of ψ can abrogate the enhancement of protein expression observed with ψ or m^1^ψ.

In accordance with previous studies, chemical modifications reduce the immunogenicity of IVT-mRNAs. However, we did not observe a correlation between the suppression of innate immunity activation and enhancement of protein expression. For example, Et^1^Ψ rendered mRNA non-immunogenic, yet it did inhibit protein expression. Early studies attributed high protein expression to reduced inhibitory effects of the cell-autonomous antiviral defense mechanisms on translation [23,24]. However, the factors that dictate how modifications not on the Watson–Crick face of nucleobases impact translation are not well understood compared to their effects on innate immune activation. Consistent with early reports, we found that nucleoside modification of IVT mRNA with ψ and m1ψ can increase the yield of protein expression, particularly seen with both natural and coding-optimized Survivin mRNA. The data obtained with GFP mRNA are comparable to those reported by Mauger et al. [34]. Indeed, no protein enhancement was seen when several Ψ- and m^1^Ψ-modified GFP sequences were tested. Also, the authors observed inhibition of translation by mo^5^U modification. More recently Mulroney et al. found that mo^5^U alone or combined with m^5^C significantly decreased translation [35]. Overall, mo^5^U does not seem to enhance protein translation to a level comparable to that achieved with ψ or m^1^ψ across several studies and mRNA sequences. Of note, protein expression in vivo can be influenced not only by RNA modifications but also by the nature of the delivery agents and the site of endogenous protein expression (e.g., liver vs. spleen) [50].

The ability of Ψ to enhance translation could be attributed to its effect on mRNA structure. Compared to uridine, Ψ has an extra hydrogen bond donor (N^1^-H^…^) in the major groove while keeping the hydrogen bond donor and acceptor as uridine in the Watson–Crick face [33,51]. The potential of ψ to modify RNA structure by enhancing base pairing, base stacking, and inducing rigidity in the phosphate backbone via its capability to coordinate a structural water molecule through N^1^-H interaction has been experimentally verified through various techniques [51,52,53]. Such structural elements were suggested to be responsible, at least in part, for evading innate immune responses and enhancing protein expression.

Among different RNA modifications, base methylation is the most common, and occurs with greater frequency and diversity in RNA compared to DNA [20,21]. Moreover, both DNA and RNA methylation silence innate immune activation [54]. Similar to pseudouridylation, methylation can also enhance RNA stability through improvement of the stacking interactions [55]. m^1^ψ has also been reported to contribute to the stability of RNA structure, despite the absence of an additional hydrogen bond donor due to the presence of a methyl group attached to the N1 position [56]. Hence, the introduction of a methyl group at the N1 position may not significantly alter the structural function of Ψ as suggested by Yarian et al. [56]. It should be noted that, despite its hydrophobic nature as an alkyl group, methylation surprisingly enhances water solubility, which has been attributed to molecular polarizability [57]. Although m^1^Ψ lacks the additional hydration site characteristic of Ψ, significant water-bridging interactions between the backbone phosphate atoms were observed within the m^1^Ψ-modified duplexes [58]. One might think that the increase in polarizability due to the presence of the methyl group could have preserved or even enhanced the primary property of Ψ as a stimulator of mRNA translation.

In addition to the biochemical properties conferred by the major groove modification as discussed above, ψ and m^1^ψ share a critical common feature, the C5-C1’ bond, which enables free rotation between the nucleobase and the sugar moiety and probably contributes to improving the RNA structure and stability through enhanced C3’ endo sugar puckering [33,53]. However, this characteristic on its own may not be sufficient for high mRNA translation. Indeed, replacement of the methyl group (-CH_3_) by a simple ethyl group (-CH_2_CH_3_) at the N1 position did not enhance protein expression; on the contrary, it severely inhibited translation, even of the coding sequence-optimized mRNA (see Figure 5C). Both methyl and ethyl groups are nonpolar, as they exclusively consist of carbon–hydrogen bonds. The main differences between a methyl group and an ethyl group in biochemical terms relate to their size and potential reactivity. In general, alkyl groups tend to donate electrons, leading to the + inductive effect (+I). Larger alkyl groups have a greater inductive (+I) effect than the CH_3_ group. It is possible that ethyl modification changes nucleobase ring electronics to perturb the strength of the hydrogen bonding interactions between mRNA codons and tRNA anti-codons. As discussed above, methylation at position N1 is more likely to induce similar structural effects as the imino group. These effects could potentially aid in directing IVT mRNA to endoplasmic reticulum-bound ribosomes, thereby resolving ribosome stalling and collisions and enhancing translation efficiency [59]. Further analysis of the crystal structures of ribosome-containing modified mRNA and tRNA should provide a molecular rationale for the inhibition of translation by Et^1^-Ψ.

In the genetic code, the first two positions of the codon-anticodon helix require Watson–Crick base pairing, while certain specific mismatches are accepted at the third (wobble) position [60]. This degeneracy in the 64 genetic codes and the limitation in the number of tRNA species require some tRNAs to read multiple codons. To resolve this problem, nature has chosen post-transcriptional modifications of tRNAs to enable one tRNA to decode cognate and “wobble” codons [60]. For instance, the incorporation of pseudouridine into anticodons could enhance the recognition of alternative codons, which might otherwise be poorly recognized during translation if the anticodons were not pseudouridylated [44]. Likewise, modifications of nucleobases within the mRNA coding regions could also influence tRNA’s ability to recognize codons. Supporting this notion, Karijolich et al. showed that substituting uridine with Ψ in stop codons impedes translational termination, both in vitro and in *Saccharomyces cerevisiae*, thereby enabling ΨAA and ΨAG to encode for serine or threonine and ΨGA to encode for phenylalanine or tyrosine [45]. Alterations to sense codons could similarly result in the production of mutant proteins. In this context, luciferase peptides derived from fully Ψ-substituted mRNAs exhibited significantly higher (over 20-fold) levels of amino acid misincorporation compared to peptides synthesized from mRNAs containing conventional uridine nucleosides [46]. In contrast to Ψ, Kim et al. showed that m^1^Ψ remains faithful in coding, more like uridine during translation [61]. Of note, the authors detected only very moderate miscoded peptides from ψ mRNA encoding the SARS-CoV-2 spike protein by LC-MS/MS analysis. However, they may have missed some peptides because they focused only on high-frequency events. Chen et al. demonstrated that T7 RNA polymerase incorporates ψ into IVT mRNA with less fidelity than m1ψ, introducing a second factor contributing to protein variability [62]. Overall, the published data suggest that ψ-modified IVT mRNA may encode a protein slightly different from that encoded by the unmodified mRNAs. Such a protein or a fraction of molecules harboring few or higher amino acid substitutions may break tolerance to self-antigens. Here, we have shown that in contrast to unmodified mRNAs, mice immunized with Ψ-modified WT1 mRNA developed an antibody response against recombinant WT1 protein prepared from HEK293T cells transfected with IVT mRNAs. Antibodies against WT1 from ψ mRNA also bound to WT1 protein from unmodified mRNA and m1ψ mRNA. This could be due to potential crossreactivity. Although further work is needed, the observed crossreactivity could be due to the phenomenon of epitope spreading [63]. In this scenario, the immune responses induced by a single peptide may spread to other peptide epitopes on the same antigen and/or to different antigens.

With respect to cancer vaccination, the use of ψ-modified IVT mRNA could be an alternative way to break self-tolerance to TAAs. Most TAAs are self-antigens and, therefore, impose potential barriers to the full development of effective immune responses against tumors. Early studies revealed that tumors with low immunogenicity, such as spontaneous tumors, can transform into highly immunogenic tumors following exposure to mutagens [64,65]. Immunization with such mutagenized tumors also induced immunity against the original non-mutagenized tumors [65]. Another strategy for overcoming immune tolerance involves inducing mutations randomly through error-prone polymerase chain reactions or utilizing altered peptide variants with single or multiple residue substitutions in potential antigenic self-peptides [66,67,68]. For example, Engelhorn and colleagues introduced mutations into the coding sequence of two syngeneic tyrosinase-related TAAs and showed that the introduced mutations could break self-tolerance and enhance anti-tumor immunity [66]. While the described strategies are promising in generating anti-tumor immunity, they are rather inefficient to induce higher protein levels that may be required for effective immunity. In contrast, the Ψ-modified IVT mRNA strategy confers high levels of protein expression (see Figure 4). The overexpression of TAAs during vaccination could expand the spectrum of self-peptides displayed by MHC molecules, surpassing the self-tolerance threshold and enabling the activation of T cells against tumors.

In addition to the mis-decoding of ψ mRNA, certain nucleotide modifications may affect the maintenance of the correct reading frame during the translation of IVT mRNAs. In this context, Mulroney et al. reported a significant increase in ribosomal +1 frameshifting during translation of m^1^ψ mRNA and noted that an immune response to +1 frameshifted products can occur following vaccination [35]. These +1 frameshifted products, containing new amino acid sequences, should be recognized by the immune system as foreign antigens and, in principle, should trigger strong T and B cell responses. Under our experimental conditions, +1 frameshifted products were likely missed during protein purification using a His-tagged strategy, which captures only in-frame products since the tag was placed at the C-terminus. In the case of Survivin, we detected only in-frame mRNA-encoded products (see Figure 4 and Figure 5). We did not detect shorter or larger potential frameshifted products in Western blots, despite the presence of some ribosome slippery sequences (e.g., ΨΨΨΨ; ΨΨΨC). This suggests that +1 ribosomal frameshifting may occur at levels too low to be detected by Western blotting. However, frameshift mutations could destroy the antigenic site, and hence antibody binding.

When comparing the pro-inflammatory responses elicited by unmodified mRNA and lipid nanoparticles or standard vaccine adjuvants, one may observe notable differences. Unmodified mRNA-induced innate immunity typically prompts mRNA degradation, while activation of innate immunity by lipid nanoparticles, for example, does not necessarily lead to mRNA degradation [69]. The primary distinction likely lies in the specific activated receptors and the subsequently activated signaling pathways. In the case of mRNA, these pathways often involve the activation of protein kinase R (PKR), which phosphorylates the eukaryotic initiation factor 2α (eIF2α), resulting in the inhibition of translation initiation [23]. Additionally, type I interferons induce the expression of 2′,5′-oligoadenylate synthetases (OAS), a group of cytoplasmic receptors, which generate 5′-phosphorylated, 2′,5′-linked oligoadenylates (2-5A) [70]. These molecules stimulate RNase L, leading to the degradation of both cellular RNAs and therapeutic mRNA [71]. Lipid nanoparticles utilized for delivering modified mRNA have their own mechanisms of immune activation, primarily via receptors that recognize the nanoparticle components rather than the mRNA itself [31]. Consequently, the pathways triggered by lipid nanoparticles or standard vaccine adjuvants may not result in the same robust inhibition of translation and/or mRNA degradation as observed with those activated by exogenous unmodified RNAs (54). For a vaccine to function properly, adjuvant activity is required. Moreover, the nature of the adjuvant will determine the type of immune response—whether it is a Th1- or Th2-type response or a mixture of responses—depending on the intended outcome.

## 5. Conclusions

The data discussed in this study reveals two significant findings. Firstly, the incorporation of Et^1^Ψ suppressed innate immune activation, but it also inhibited IVT mRNA translation, unlike Ψ or m^1^Ψ. This suggests that the C5-C1’ bond, a major intrinsic characteristic of pseudouridylation, alone is not responsible for high mRNA translation. The fact that translation can be inhibited by adding an ethyl group at the N1 position of ψ suggests that there are additional structural constraints on which modified nucleosides can be recognized by the ribosome. Secondly, vaccination with ψ-modified WT1 mRNA induced an antibody response against WT1 protein, whereas vaccination with unmodified WT1 mRNA did not. Given that WT1 is a self-antigen in this context, this observation could pave the way for the induction of immunity against TAAs, many of which are self-proteins. Exploring potential defects in translation fidelity due to RNA modifications or cellular stress responses, which may disrupt tolerance to TAAs and/or generate neoantigens, could broaden the scope of immunotherapy targets and improve immunotherapy approaches.

## Data Availability

All data described in this study are included in the figures of the manuscript. Recombinant plasmids are available from the corresponding author upon request.

## Supplementary Materials

The following supporting information can be downloaded at: https://www.mdpi.com/article/10.3390/vaccines12060624/s1

## Abbreviations

ELISA	Enzyme-linked immunosorbent assay
GFP	Green Fluorescent protein
hm^5^U	5-hydroxymethyluridine
IVT	In vitro transcription
IFN-α	Interferon alpha
mo^5^U	5-methoxyuridine
m^5^U	5-methyluridine
Et1ψ	N1-ethylpseudouridine
m^1^ψ	N1-methylpseudouridine
ψ	Pseudouridine
siRNA	Small interfering RNA
TAAs	Tumor-associated antigens
TLR	Toll-like receptor
TNF-α	Tumor necrosis factor-alpha
U	Uridine
WT1	Wilms’ Tumor Antigen-1
