# Supplementary material for: Exploring the Impact of mRNA Modifications on Translation Efficiency and Immune Tolerance to Self-Antigens
Source: Vaccines (Basel). 2024 Jun 5;12(6):624. doi: 10.3390/vaccines12060624 (PMC11209393; doi:10.3390/vaccines12060624)
Supplement: Supplementary file 1 [file vaccines-12-00624-s001.zip › vaccines-3022321-supplementary.pdf]

Sequences of the open reading frames of *in-vitro* transcribed RNA molecules are as follow:
During transcription, all uridines (shown as T) were replaced by either ψ, $m^1$ψ, $Et^1$ψ, $m^5U$, $mo^5U$, or $hm^5U$.

- GFP open reading frame

**ATG**GTGAGCAAGGGCGAGGAGCTGTTCACCGGGGTGGTGCCCATCCTGGTCGAGCTGGACGGCGACGTAAA
CGGCCACAAGTTCAGCGTGTCCGGCGAGGGCGAGGGCGATGCCACCTACGGCAAGCTGACCCTGAAGTTCAT
CTGCACCACCGGCAAGCTGCCCGTGCCCTGGCCCACCCTCGTGACCACCCTGACCTACGGCGTGCAGTGCTTCA
GCCGCTACCCCGACCACATGAAGCAGCACGACTTCTTCAAGTCCGCCATGCCCGAAGGCTACGTCCAGGAGCG
CACCATCTTCTTCAAGGACGACGGCAACTACAAGACCCGCGCCGAGGTGAAGTTCGAGGGCGACACCCTGGT
GAACCGCATCGAGCTGAAGGGCATCGACTTCAAGGAGGACGGCAACATCCTGGGGCACAAGCTGGAGTACAA
CTACAACAGCCACAACGTCTATATCATGGCCGACAAGCAGAAGAACGGCATCAAGGTGAACTTCAAGATCCGC
CACAACATCGAGGACGGCAGCGTGCAGCTCGCCGACCACTACCAGCAGAACACCCCCATCGGCGACGGCCCC
GTGCTGCTGCCCGACAACCACTACCTGAGCACCCAGTCCGCCCTGAGCAAAGACCCCAACGAGAAGCGCGATC
ACATGGTCCTGCTGGAGTTCGTGACCGCCGCCGGGATCACTCTCGGCATGGACGAGCTGTACAAGTAAAGCG
GCCGCTTCGAGCAGACA**TGATAA**

- Survivin Open reading frame

**ATG**GGTGCCCC GACGTTGCCC CCTGCCTGGC AGCCCTTTCT CAAGGACCAC CGCATCTCTACATTCAAGAA
CTGGCCCTTC TTGGAGGGCT GCGCCTGCAC CCCGGAGCGG ATGGCCGAGGCTGGCTTCAT CCACTGCCCC
ACTGAGAACG AGCCAGACTT GGCCCAGTGT TTCTTCTGCT TCAAGGAGCT GGAAGGCTGG GAGCCAGATG
ACGACCCCAT AGAGGAACAT AAAAAGCATT CGTCCGGTTG CGCTTTCCTT TCTGTCAAGA AGCAGTTTGA
AGAATTAACC CTTGGTGAAT TTTTGAAACT GGACAGAGAA AGAGCCAAGA ACAAAATTGC AAAGGAAACC
AACAATAAGA AGAAAGAATT TGAGGAAACT GCGAAGAAAG TGCGCCGTGC CATCGAGCAG CTGGCTG C
CATGGAT**TGA**

- Survivin Open reading frame (codon sequence optimized)

**ATG**GGCGCCCCGACACTGCCGCCAGCCTGGCAGCCCTTCCTGAAGGACCACAGAATCTCCACCTTCAAGAACT
GGCCCTTCCTGGAAGGCTGCGCCTGCACCCCCGAGAGAATGGCCGAGGCCGGCTTCATCCACTGCCCCACCGA
GAACGAGCCCGACCTGGCCCAATGTTTCTTCTGCTTCAAGGAGCTGGAAGGCTGGGAGCCCGACGACGACCCC
ATCGAGGAACACAAGAAGCACAGCAGCGGATGCGCCTTCCTGAGCGTGAAGAAGCAGTTCGAGGAACTGACC
CTGGGCGAGTTCCTGAAGCTGGACAGAGAGCGGGCCAAGAACAAGATCGCCAAAGAGACAAACAACAAGAA
GAAGGAGTTCGAGGAAACCGCCAAGAAAGTGCGGAGAGCCATCGAGCAGCTGGCTGCCATGGAC**TGA**

- Mouse Wilms' tumor antigen 1 open reading frame

**ATG**GGCAGCGACGTGCGGGACCTGAACGCCCTGCTGCCTGCCGTGTCTTCTCTGGGCGGAGGCGGAGGAGGC
TGCGGCCTGCCTGTGTCTGGCGCTCGGCAGTGGGCCCCTGTGCTGGATTTTGCCCCTCCTGGAGCTAGCGCCT
ACGGCTCTCTGGGAGGACCTGCCCCTCCTCCTGCCCCACCACCTCCTCCACCTCCACCCCACAGCTTCATCAAGC
AGGAACCTAGCTGGGGCGGAGCCGAGCCTCACGAGGAACAGTGCCTGAGCGCCTTCACCCTGCACTTCAGCG
GCCAGTTCACCGGCACCGCTGGCGCCTGTAGATACGGCCCTTTCGGCCCTCCACCACCCTCTCAGGCTAGCTCT
GGCCAGGCTAGAATGTTCCCCAACGCCCCCTACCTGCCCAGCTGCCTGGAAAGCCAGCCCACCATCCGGAACC
AGGGCTACAGCACCGTGACCTTCGACGGCGCCCCCAGCTACGGCCACACCCCTTCTCACCACGCCGCCCAGTTC
CCCAACCACTCCTTCAAGCACGAGGACCCCATGGGCCAGCAGGGCAGCCTGGGCGAGCAGCAGTACAGCGTG
CCCCCTCCTGTGTACGGCTGCCATACCCCCACCGATAGCTGCACAGGCAGCCAGGCCCTGCTGCTGAGAACCCC
CTACAGCAGCGACAACCTGTACCAGATGACCAGCCAGCTGGAATGCATGACCTGGAACCAGATGAACCTGGG
AGCCACCCTGAAGGGCCACGGCATCGGCTACGAGAGCGAGAACCACACCGCCCCCATCCTGTGTGGCGCCCA
GTACCGGATCCACACCCACGGCGTGTTCCGGGGCATCCAGGACGTGCGAAGAGTGTCCGGCGTGGCCCCTAC
ACTCGTGCGGAGCGCCAGCGAGACAAGCGAGAAGCGGCCCTTCATGTGCGCCTACCCCGGCTGCAACAAGCG
GTACTTCAAGCTGAGCCACCTCCAGATGCACAGCCGGAAGCACACCGGCGAGAAGCCCTACCAGTGCGACTTC
AAGGACTGCGAGCGGCGGTTCAGCAGAAGCGACCAGCTGAAGCGGCACCAGCGGAGACACACAGGCGTGAA

GCCTTTTCAGTGCAAGACCTGCCAGCGGAAGTTCAGCCGCTCCGACCACCTGAAAACCCACACCCGGACCCAC
ACAGGGGAGAAGCCATTCAGCTGCCGGTGGCACTCCTGCCAGAAGAAGTTCGCCCGCAGCGACGAACTCGTG
CGGCACCACAACATGCACCAGAGAAACATGACCAAGCTCCACGTGGCCCTC<u>CACCACCACCACCACCAC</u>**TGA**

The sequence coding for the 6x His tag is underlined
